# Epigenetic targeting to enhance acute myeloid leukemia-directed immunotherapy

**DOI:** 10.3389/fimmu.2023.1269012

**Published:** 2023-09-22

**Authors:** Johanna Rausch, Evelyn Ullrich, Michael W.M. Kühn

**Affiliations:** ^1^ Department of Hematology and Medical Oncology, University Medical Center, Johannes Gutenberg-University, Mainz, Germany; ^2^ German Cancer Consortium (DKTK) Partner Site Frankfurt/Mainz and German Cancer Research Center (DKFZ), Heidelberg, Germany; ^3^ Children’s Hospital, Experimental Immunology, Johann Wolfgang Goethe University, Frankfurt, Germany; ^4^ Frankfurt Cancer Institute, Goethe University, Frankfurt, Germany; ^5^ University Cancer Center (UCT), Frankfurt, Germany

**Keywords:** acute myeloid leukemia, hypomethylating agents, immunotherapy, epigenetics, checkpoint inhibition, cellular therapy, chromatin modifiers, combination therapy

## Abstract

AML is a malignant disease of hematopoietic progenitor cells with unsatisfactory treatment outcome, especially in patients that are ineligible for intensive chemotherapy. Immunotherapy, comprising checkpoint inhibition, T-cell engaging antibody constructs, and cellular therapies, has dramatically improved the outcome of patients with solid tumors and lymphatic neoplasms. In AML, these approaches have been far less successful. Discussed reasons are the relatively low mutational burden of AML blasts and the difficulty in defining AML-specific antigens not expressed on hematopoietic progenitor cells. On the other hand, epigenetic dysregulation is an essential driver of leukemogenesis, and non-selective hypomethylating agents (HMAs) are the current backbone of non-intensive treatment. The first clinical trials that evaluated whether HMAs may improve immune checkpoint inhibitors’ efficacy showed modest efficacy except for the anti-CD47 antibody that was substantially more efficient against AML when combined with azacitidine. Combining bispecific antibodies or cellular treatments with HMAs is subject to ongoing clinical investigation, and efficacy data are awaited shortly. More selective second-generation inhibitors targeting specific chromatin regulators have demonstrated promising preclinical activity against AML and are currently evaluated in clinical trials. These drugs that commonly cause leukemia cell differentiation potentially sensitize AML to immune-based treatments by co-regulating immune checkpoints, providing a pro-inflammatory environment, and inducing (neo)-antigen expression. Combining selective targeted epigenetic drugs with (cellular) immunotherapy is, therefore, a promising approach to avoid unintended effects and augment efficacy. Future studies will provide detailed information on how these compounds influence specific immune functions that may enable translation into clinical assessment.

## Introduction

Acute myeloid leukemia (AML) is a malignant neoplasm of hematopoietic progenitor cells driven by acquired genetic aberrations that mediate uncontrolled proliferation and a block in differentiation (1, 2).

Novel mechanism-based drugs have improved treatment options in recent years (2), but intensive chemotherapy is still the backbone of curative treatment and induces complete remissions in up to 70% of patients (3). However, relapse is common, and overall survival is generally unsatisfactory and heterogeneous based on two significant factors: the genetic alterations of individual AML blasts and the patient’s age at diagnosis (2, 4). Despite intensive treatment, most elderly patients will ultimately succumb to their disease (2–5). Survival for patients unfit for intensive treatment is dismal, with a 5-year overall survival (OS) below 10% with current standard of care options (3–6) underpinning the need for more efficient and less toxic treatment options.

Epigenetic dysregulation has been recognized as an essential driver for leukemogenesis, thereby providing a therapeutic opportunity. Hypomethylating agents (HMA) are non-selective first-generation epigenetic drugs and are considered a mainstay in treating unfit and elderly patients (7). Several more selective compounds targeting specific epigenetic dependencies have been developed in recent years with promising responses in clinical trials (8–10). Immunotherapy has revolutionized the treatment of solid tumors and lymphatic neoplasms (11–30), but has been far less successful against AML. Mechanisms behind the limited efficacy remain obscure but have been attributed to difficulties in finding a target exclusively expressed on AML blasts, their relatively low mutational burden, and low neo-antigen expression (31–34).

Epigenetic manipulation has been reported to induce immune modulatory effects, including an increased expression of tumor-associated antigens (35, 36) that may sensitize AML blasts for immunotherapy. Here we review the concept of combined epigenetic targeting with immunotherapeutic approaches against AML.

## Epigenetic treatment in AML

Epigenetic dysregulation has been implicated in the pathogenesis of most cancer types, including AML. Sequencing efforts to characterize the genomic landscape of various cancer types have revealed recurrent mutations in epigenetic regulators, affecting AML in more than 60% of cases (37, 38). Epigenetic regulators determine the chromatin state by controlling regulatory regions and gene expression via chemical modifications, including DNA methylation and histone protein acetylation, methylation, or phosphorylation as reviewed elsewhere (39–41). Therefore, epigenetic regulators were recognized as therapeutic opportunities for many cancers, particularly AML.

First-generation HMAs such as azacitidine and decitabine are non-selective drugs that reduce promotor hypermethylation to restore the expression of tumor suppressor genes (42). These drugs have built the backbone for non-intensive AML treatment (7), and their combination with the BCL2 inhibitor venetoclax is the current standard of care for unfit AML patients resulting in a median overall survival (OS) of 14.7 months (6). Histone deacetylase (HDAC) inhibitors, another class of non-selective epigenetic drugs that initially showed promising activity in preclinical models (43), failed to induce sustainable remissions in clinical trials in monotherapy (44–46). Reasons for the low efficacy in clinical studies are not fully elucidated, however missing predictive biomarkers, the heterogeneous activity of different HDAC inhibitors, and dose-limiting off-target effects of pan-HDAC inhibitors remain an unsolved problem, especially in combination with other anti-neoplastic agents (47–49).

Second-generation epigenetic inhibitors were developed to target specific chromatin modifiers and epigenetic dependencies in various cancers with potentially less off-target toxicity. Research has particularly focused on the development and clinical assessment of drugs targeting the following chromatin modifiers:

Bromodomain-containing transcriptional activators (BRDs) are recruited to histone-acetylated transcription sites to accelerate gene expression. BRD4 is a Bromodomain and extra-terminal (BET) protein, and its function is best characterized in AML (50, 51). Inhibitors of BET proteins, particularly BRD4, have shown promising preclinical activity (52) but demonstrated only modest activity as a single agent against AML with an overall response rate (ORR) of only 6% in relapsed refractory (R/R) AML (53).

The histone methyltransferase Disruptor of Telomeric Silencing 1-like (DOT1L) is the only histone 3 lysine 79 methyltransferase known to date. It maintains leukemic transcription in leukemias with Mixed-Lineage Leukemia (MLL, also known as KMT2A)-rearrangement (*MLL*-r) or partial tandem duplication and NPM1 mutant (*NPM1* ^mut^ *)* leukemia (54, 55). Similar to BET inhibitors, the first clinical trials with DOT1L inhibitors demonstrated limited activity with only two complete remissions (CR) in 52 patients in a phase I trial (56) despite promising preclinical activity (54, 55).

Protein Arginine Methyltransferase 5 (PRMT5) regulates gene expression by dimethylation of histone and non-histone proteins (e.g.,RNA splicing factors) (57, 58). Inhibition of PRMT5 has demonstrated anti-leukemic activity and induction of differentiation in preclinical *MLL*-r and *FLT3*-ITD AML models (59, 60), and several inhibitors are currently evaluated in early clinical trials for solid tumors, lymphomas, and leukemias, which was reviewed elsewhere (61). In brief, phase I studies have reported limited efficacy, with common adverse effects in solid tumors and primary myelofibrosis (62–64). One phase I study is currently recruiting AML patients (65).

Enhancer of Zeste Homolog 2 (EZH2) is a lysine methyltransferase and the catalytic subunit of Polycomb Repressive Complex 2 (PRC2) that silences its target genes via H3K27 trimethylation (66, 67). EZH2 mutations are found in solid tumors and usually as gain-of-function events in lymphomas (68, 69). The inhibitor tazometestat induced durable and complete responses in Phase I/II trials in sarcomas and lymphomas (70–72). EZH2 has been reported to act context-dependently as a tumor suppressor or sometimes as an oncogene in myeloid malignancies (66, 73). Its loss has been associated with poor prognosis and chemotherapy resistance, and mutations are more common in relapsed AML patients (74–76). EZH1/2 inhibition has demonstrated *in vitro* and *in vivo* anti-leukemic activity (77, 78). Clinical outcome data for EZH2 inhibition in AML do not exist, also because a phase I trial was terminated due to insufficient patient recruitment (NCT03110354).

Lysine-Specific Demethylase-1 (LSD1, also known as KDM1A) is a histone 3 demethylase and is believed to participate in the control of leukemic gene expression programs (79). LSD1 inhibition had promising activity in preclinical leukemia models, and preliminary efficacy against AML has been reported from an ongoing clinical phase I/II trial (80, 81). Additional studies are needed to define the clinical activity in specific AML subtypes in detail.

Dramatic clinical responses in AML were observed with specific inhibitors of mutant isocitrate-dehydrogenase (IDH) 1 and 2 enzymes and are also explained by epigenetic mechanisms: Mutations in IDH1 and IDH2 lead to a neo-enzyme activity of both enzymes, accumulating the ordinarily absent oncometabolite 2-hydroxyglutarate (2-HG) (82). 2-HG inhibits ten-eleven translocation (TET) family enzymes responsible for DNA methylation, ultimately resulting in aberrant expression of leukemic genes (83). IDH1/2 inhibition induces cell differentiation of IDH-mutated AML blasts (84). The first phase I trial assessed the IDH2 inhibitor enasidenib as a single agent with an ORR of 40.3% and a median OS rate of 9.3 months in R/R AML patients (85). The combination of the IDH1 inhibitor ivosidenib with azacitidine was recently approved for newly diagnosed *IDH1* mutated AML in Europe and the U.S. The approval was based on a randomized, placebo-controlled phase III trial where the combination significantly increased CR rates (47% vs. 15%, p<0.001) and survival (recently updated median OS: 29.3 vs. 7.9 months; HR 0.42, p-value <0.0001) compared to azacitidine plus placebo (9, 86).

A novel epigenetic target and auspicious therapeutic opportunity against specific AML subtypes is the protein interaction of the histone methyltransferase KMT2A (also known as MLL1) with its oncogenic adaptor protein menin (encoded by the *MEN1* gene). While it was reported that menin is required for chromatin binding and target gene activation of oncogenic MLL1-fusion proteins in *MLL1*-rearranged leukemias (87), our group reported that the direct interaction of wildtype MLL with menin is a dependency in the most prevalent *NPM1* ^mut^ AML subtype (55). Characteristic leukemic gene expression programs, including high-level expression of *MEIS1*, *PBX3*, and various *HOX* transcription factor genes, also depend on the protein interaction (55). Pharmacological inhibition of the menin-MLL interaction has demonstrated profound *in vitro* and *in vivo* anti-leukemic activity inducing uniform transcriptional repression of *MEIS1*, *PBX3*, *FLT3*, and *BCL2*, and leading to differentiation and apoptosis in *MLL*-r and *NPM1* ^mut^ leukemias (55, 87–90). These preclinical data translated into an ongoing clinical assessment of five different menin inhibitors against AML (NCT04067336, NCT04065399, NCT05153330, NCT04811560, NCT04988555) with astonishing first efficacy data from two phase I trials: The oral menin inhibitor revumenib induced complete remissions (combined; CRc) in 38% of heavily pretreated R/R AML with *NPM1* ^mut^ or *MLL*-r as a single agent, with responding patients exhibiting sustainable responses of more than 9.1 months (8). Ziftomenib also had promising clinical activity in *NPM1* ^mut^ or *MLL*-r R/R AML, with 35% of patients achieving CR/CRh or CRp rate in a phase I/II study (91). The single-agent evaluation of both drugs is currently ongoing. Combinatorial clinical trial assessment with intensive chemotherapy and specific small molecule inhibitors is also underway, as both inhibitors have exhibited synergistic *in vitro* and *in vivo* efficacy with various targeted cancer drugs (92–94).

## Targeting the immune system in AML

Within the last decade, similarly great excitement has greeted cancer immunotherapy, revolutionizing the treatment of many cancer types (11–30). Concepts to guide the immune system in recognizing and fighting cancer cells comprise antibody-directed targeting, blockage of immune checkpoints, and adoptive transfer of immune cells. These approaches have led to sustainable responses, prolonged survival, and even cure of previously untreatable malignancies, but single-agent efficacy against AML has been limited.

Immune checkpoint blockade (ICB) with anti-CTLA-4 and anti-PD-L1/PD-1 antibodies dramatically improved overall survival in patients with advanced solid tumors as well as Hodgkin’s lymphoma (13–18) and is now considered the standard of care for the treatment of many other cancer entities.

AML cells also have higher surface expression of inhibitory immune checkpoints (such as PD-L1) compared to normal hematopoietic stem (HSCs) and progenitor cells (HSPCs) and higher expression of PD-1 is observed on T-cells of AML patients compared to healthy donors (95–102). Still, clinical trials assessing therapeutic checkpoint blockade yielded generally discouraging results in myeloid neoplasms. Only 1 out of 9 patients with AML or myelodysplastic syndrome (MDS) responded to the anti-PD-1 antibody pidilizumab in a first phase I trial (103). Also, ORR in studies assessing the anti-PD-1 antibody pembrolizumab and anti-PD-L1 antibody atezolizumab in R/R MDS patients were only 4% and 0%, respectively (104, 105). Responses to the anti-CTLA-4 antibody ipilimumab in early clinical trials assessing selected AML patients that relapsed following allogenic stem cell transplantation (SCT) were more promising, with 23% of patients achieving a CR. However, treatment was commonly associated with severe graft versus host disease (12).

CD47 is a checkpoint of the innate immune system that mediates a “do not eat me “ signal to macrophages (106, 107). Magrolimab, a monoclonal anti-CD47 antibody, demonstrated limited efficacy as a single-agent in AML with no objective responses (stable disease: 73%) (108), but might be more efficacious if added to established combination regimens (discussed below).

Bispecific T-cell engager (BiTE) or dual-affinity retargeting antibodies (DART) are artificial antibody constructs that contain two antigen binding sites, one directed against immune effector cells (mostly CD3 for T-cells) and the other against a specific surface antigen on tumor cells. The convergence leads to T- or NK-cell activation and killing (31). BiTEs targeting CD3 and CD19, such as blinatumomab, are efficient against and approved for treating B-cell neoplasms (28). Defining a unique leukemic target on myeloid blasts has yet limited efforts to extend this concept for successful AML treatment (discussed below), and so far, efficacy has been unsatisfactory. In a phase I trial assessing the anti-CD33xCD3 directed bispecific antibody AMG330 against R/R AML, CR/CRi rates were 17% (109) and 3 and 5% in ongoing phase I studies testing the anti-CD33xCD3 BiTE molecules AMV564 and AMG673 (110, 111). Reported ORR from a phase I/II trial exploring flotetuzumab, an anti-CD123xCD3 DART construct, against R/R AML was 30%. However, treatment was associated with high rates of severe cytokine release syndrome (CRS) (81%, 8% ≥3) (112), which was also commonly observed with the bispecific anti-CD123 antibody XmAb14045 (113). Other CD123-targeting antibodies are under clinical investigation (NCT03647800, NCT02715011).

Several reports suggest that the myeloid antigens WT1, PRAME, and CLL-1 (CLEC12A) are expressed only at low levels on HSCs, which may be associated with less hematologic toxicity if targeted by immunotherapy (32, 114–117). A lower CRS rate was reported from a phase I trial exploring the first CLL-1xCD3-directed bispecific antibody MCLA-117 in R/R AML but with only 15% of patients achieving a partial response (118).

Cellular immunotherapy describes the adoptive transfer of genetically engineered autologous chimeric-antigen receptor (CAR)-T or -Natural Killer (NK) cells. Astonishing successes were reported from treatment of B-cell neoplasms with various CAR-T cell products and have led to their approval in the Europe and the U.S. (19–28). As with BITEs and DARTs, CAR construct development against AML faces similar challenges in defining unique immunotargets on AML blasts. Lineage-specific antigens such as CD33 and CD123 are commonly expressed on AML blasts and evaluated as potential targets. Their expression on hematologic stem cells (HSCs) bears the risk of post-treatment bone marrow failure (32, 119, 120). As CAR-T cells commonly have a “memory effect”, hematologic toxicity might be even more severe compared to BITEs and DARTs.

One strategy to avoid the off-tumor toxicity is the development of AND-gated and NOT-gated CAR-T cells that engage two antigens to increase selectivity (121, 122). Perriello et al. developed cytokine-induced killer (CIK) cells with two CARs directed against CD123 and CD33. In this case, simultaneous binding of both CARs is necessary for a cytotoxic T-cell activation, because the CD33 CAR delivers the essential co-stimulatory signal (122). The authors also demonstrate that reduced binding activity of a CAR may increase selectivity by restricting reactivity to cells with high antigen expression. NOT-gates CARs represent an different approach to avoid off-tumor toxicity: Richards et al. developed CD93-directed CAR T-cells that express a second inhibitory CAR (iCAR) directed against an antigen present on endothelial cells but absent on myeloid blasts. This iCAR contains endodomains from ITIM-containing proteins including PD-1, TIM-3 or TIGIT delivering an inhibitory signal that interferes with the CAR T-cell activation signal (121).

So far, CAR-T-cells targeting CD33, CD123, or two antigens at once (e.g., CD33 and CLL-1; CD13 and TIM-3) are currently evaluated in early clinical trials (NCT03971799, NCT03795779, NCT03631576, NCT03190278, NCT03114670, NCT02159495, NCT04272125, NCT03222674, NCT04010877, NCT04097301). Three studies reported activity against heavily pretreated patients (123–125), but longer follow-up efficacy data needed to draw more definitive conclusions are pending. For CD70, another immune target expressed on AML blast and low expression on HSCs, promising activity has been reported in preclinical AML models. Clinical trial evaluation is expected shortly (126, 127).

CAR-engineered NK cells may have potential advantages over CAR-T cells and be a promising alternative for two reasons: a) their HLA-class I independent tumor cell recognition allows maintaining intrinsic anti-tumor activity in case of antigen loss (128), and b) the lack of clonal expansion protects recipients from persistent graft versus host disease (GvHD) or long-term hematologic toxicity, reviewed in (129). First clinical applications have demonstrated encouraging anti-leukemic activity and tolerability with cord-blood-derived CD19-CAR NK cells against chronic lymphatic leukemia (130). CAR-NK cell products are effective against preclinical AML models *in vitro* and *in vivo* but clinical activity remains to be demonstrated (131).

While the efficacy of these concepts still needs improvement, the strong graft versus leukemia effect that has been observed over decades following allogenic SCT indicates that AML may still be prone to immunotherapy (132–134). As mentioned above, one potential reason might be the particularly low mutational burden found in AML blasts compared to other cancers, which has been associated with generally lower responses to immune-based treatments (33, 34). Defining an AML-specific immunotarget that is not expressed on HSC is also an ongoing challenge for the development of potent immune-based treatments (32).

## Combination of epigenetic treatment with immunotherapy

**Figure 1:**
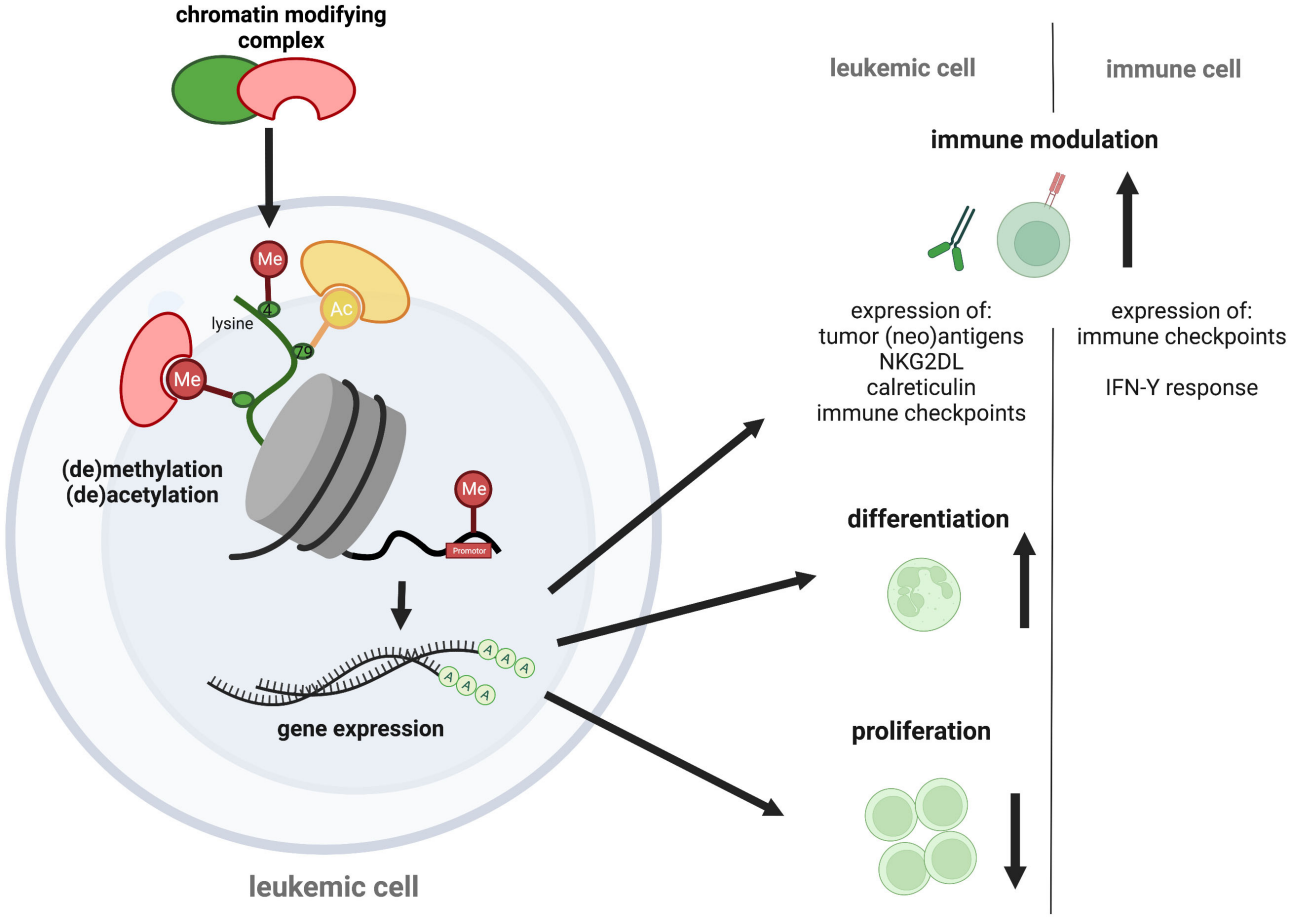
Epigenetic targeting in AML. Epigenetic regulators control transcription via chemical chromatin modifications, including histone protein and DNA (de-)methylation or histone (de-)acetylation that determine chromatin state. As therapeutic opportunities against AML, chromatin modifiers can alter leukemogenic gene expression, causing cell differentiation and proliferation arrest of the malignant blasts. Additional pro-immunogenic effects have recently been discussed, including an increased neoantigen-, immune checkpoint-, NK2GDL- and calreticulin expression on leukemic blasts and an augmented immune checkpoint expression and IFN-Y response of immune cells. The figure was created with BioRender.com.

Epigenetic mechanisms have been implicated in contributing to the poor responses of AML to immunotherapy. One example is the silencing of HLA class II molecules observed in AML patients that relapsed after allogenic SCT (135–137). This has been attributed to the DNA-hypermethylation of respective promotor regions (96). Therapeutic manipulation with HMAs to reverse promotor-methylation has successfully been used at relapse to boost graft-versus leukemia effects of donor lymphocyte infusions. However, this concept is less efficient with high leukemia burden (138–140). Additional immune modulatory effects of HMA are currently being discussed. These include enhanced expression of tumor-associated antigens such as MAGE-1 and NY-ESO-1 (35, 36). Also, HMA-treatment is associated with tumor re-expression of endogenous retroviruses (ERVs) that is believed to improve T- and NK-cell activation via enhanced IFN-γ expression (141–144), enhances tumor lymphocyte infiltration (145), and impairs expansion of regulatory T-cells (146), (**Figure 1**). The limited activity of HMAs commonly observed in the clinical setting may partly be explained by the upregulation of the immune inhibitory checkpoints (147, 148).

HMA treatment has also been investigated in combination with immune checkpoint blockade in clinical trials. Encouraging results demonstrated a first phase II trial assessing the combination of PD-1 antibody nivolumab and azacitidine in R/R AML resulting in an ORR of 58% in HMA-naive and 22% in HMA-pretreated patients, respectively (149). Newly diagnosed and R/R patients achieved a CRc in 47% and 14% in a phase II trial assessing the combination of the PD-1 antibody pembrolizumab with azacitidine (150). Azacitidine combined with the anti-TIM-3 monoclonal antibody sabatolimab led to an ORR of 57% and a CRc of 30% in newly diagnosed AML in a phase Ib trial (151). The only randomized data available come from a trial assessing the anti-PD-L1 antibody durvalumab, Here, no significant benefit for the combination of durvalumab and azacitidine was observed over azacitidine alone in MDS/AML patients (152). Consistent with the data above, the authors of a recent meta-analysis concluded that the activity of checkpoint inhibitors is generally low in the relapsed/refractory AML setting (153). Further studies are currently ongoing (**Table 1**).

**Table 1:** Current clinical trials evaluating combinations of epigenetic targeting and immunotherapy in AML.

NCT Trial	Patient Eligibility	Drug Combination	Clinical Phase	Status
HMA + PD1				
NCT02845297	R/R, ND elderly/unfit	Pembrolizumab + Azacitidine	Phase II	completed
NCT02397720	R/R, ND elderly/unfit	Nivolumab + Azacitidine +/- Ipilimumab	Phase II	recruiting
NCT03825367	R/R, pediatric	Nivolumab + Azacitidine	Phase I/II	active, not recruiting
NCT03769532	MRD relapse in NPM1 mut.	Pembrolizumab + Azacitidine	Phase II	recruiting
NCT02996474	R/R	Pembrolizumab + Decitabine	Phase I/II	completed
NCT03969446	R/R, ND elderly/unfit	Pembrolizumab + Decitabine +/- Venetoclax	Phase I	recruiting
NCT04284787	ND elderly/unfit	Azacitidine + Venetoclax +/- Pembrolizumab	Phase II, randomized	active, not recruiting
NCT04277442	ND, TP53 mut.	Nivomumab + Decitabine + Venetoclax	Phase I	active, not recruiting
NCT03358719	ND + R/R	NY-ESO-1 vaccination + Decitabine + Nivolumab	Phase I	completed
NCT04722952	R/R	Visilizumab + Azacitidine + Homoharringtonine, Cytarabine (HAG)	Phase III	recruiting
NCT05772273	R/R post aHSCT	Camrelizumab + Azacitidine + Low-dose DLI	–	recruiting
NCT03092674	ND elderly/unfit	Azacitidine +/- Nivolumab or Midostaurin vs. Decitabine + Cytarabine	Phase II/III, randomized	active, not recruiting
HMA + PD-L1				
NCT02775903	ND elderly/unfit	Azacitidine +/- Durvalumab	Phase II, randomized	completed
NCT02281084	R/R to HMA	CC-486 +/- Durvalumab	Phase II, randomized	active/not recruiting
NCT02953561	R/R	Avelumab + Azacitidine	Phase I/II	terminated
NCT02892318	R/R, ND elderly/unfit	Atezolizumab + Guadecitabine	Phase I	completed
NCT02935361	R/R	Atezolizumab + Guadecitabine	PhaseI/II	active, not recruiting
NCT03395873	ND elderly/unfit	Avelumab + Decitabine	Phase I	terminated (AZA/VEN approval)
NCT03390296	R/R	Poly-chemotherapy combinations of OX40, Venetoclax, Avelumab, Glasdegib, Gemtuzumab Ozogamicin, and Azacitidine	Phase I/II	completed
HMA + TIM-3				
NCT04623216	MRD positive post aHSCT	Sabatolimumab +/- Azacitidine	Phase I/II	recruiting
NCT04150029	ND elderly/unfit	Sabatolimumab + Azacitidine + Venetoclax	Phase II	active, not recruiting
NCT03066648	R/R, ND elderly/unfit	Sabatolimumab +/- Decitabine +/- Spartalizumab vs. Azacitidine + Sabatolimumab	Phase I	active, not recruiting
NCT05367401	R/R, ND elderly/unfit	Sabatolimumab + Magrolimab +/- Azactidine	Phase I/II	not yet recruiting
NCT05426798	R/R, ND elderly/unfit	TQB2618 + Azacitidine/Decitabine	Phase I	recruiting
NCT05367401	R/R, ND elderly/unfit	Sabatolimab + Magrolimab + Azacitidine	Phase I/II	not yet recruiting
HMA + CTLA-4				
NCT02890329	R/R	Ipilimumab + Decitabine	Phase I	active, not recruiting
NCT02397720	R/R, ND elderly/unfit	Nivolumab + Azaztidine +/- Ipilimumab	Phase II	recruiting
HMA + LAG3 + PD-1				
NCT04913922	R/R, ND elderly/unfit	Nivolumab + Relatlimab + Azacitidine	Phase II	recruiting
IDH1 + PD-1				
NCT04044209	R/R	IDH1 + Nivolumab	Phase II	withdrawn, no patient recruitment
HMA + CD47				
NCT05823480	after HCT	Magrolimab + Azacitidine	Phase I	not yet recruiting
NCT05367401	RR, ND elderly/unfit	Magrolimab + Azacitidine + Sabatolimumab	Phase I/II	not yet recruiting
NCT05079230	ND elderly/unfit	Azacitidine + Venetoclax + Magrolimab vs. Placebo	Phase III, randomized	recruiting
NCT04435691	R/R, ND elderly/unfit	Magrolimab + Azacitidine + Venetoclax	Phase I/II	recruiting
NCT04778397	ND with TP53 mut.	Magrolimab + Azacitidine + Venetoclax vs. Physician’s Choice	Phase III	recruiting
NCT02472145 trial in the HMA + CD123				
NCT04086264	R/R, ND elderly/unfit	IMGN632 +/- Azacitidine +/- Venetoclax	Phase I/II	recruiting
NCT02472145	ND elderly/unfit, R/R	Talacotuzumab (CD123/CD16) + Decitabine vs. Decitabine, randomized	Phase II/III	completed
HMA + CD70				
NCT03030612	ND elderly/unfit	Cusatuzumab + Azacitidine	Phase I/II	completed
NCT04227847	R/R	SEA-CD70 +/- Azacitidine	Phase I	recruiting
NCT04150887	ND elderly/unfit	Cusatuzumab + Venetoclax +/- Azacitidine	Phase I	active not recruiting
HMA + NK-cell therapy				
NCT05834244	R/R	allogeneic NK + Azacitidine + Venetoclax	Phase I	not yet recruiting

HMAs in combination with immune checkpoint inhibitors were also assessed in the post-transplant setting, with only a few responses reported and increased immune-related toxicity (12, 154). This was demonstrated by the combination of avelumab and azacitidine, resulting in CR rates of only 10.5% and an increased risk of severe graft versus host disease (155). Several clinical trials are ongoing and will allow more definitive conclusions concerning efficacy and safety.

HDAC inhibitors can also induce tumor-associated antigens, improve antigen presentation, influence T-cell trafficking and activity but also increase PD-1 expression (156–159). Several trials reported responses to HDAC inhibitors in combination with checkpoint blockade in solid tumors (160). However, in R/R MDS/AML patients, no activity of this concept has been reported in a recent phase 1b study assessing pembrolizumab plus entinostat with no responses in any of the patients (161).

In contrast, encouraging activity of combining the anti-CD47 antibody magrolimab with azacitidine and the BCL2-inhibitor venetoclax was reported from a phase I/II trial in the adverse *TP53* mutated AML subtype. CRc rates were 63%, with an average one-year overall survival of 53% (162). Two randomized phase III trials are currently ongoing (NCT05079230, NCT04778397, **Table 1**).

HMAs and HDAC inhibitors were also reported to increase the expression of AML-associated antigens such as CD33 (163) and may therefore be a suitable combination partner for BiTEs, DARTs, and CAR-T, and -NK-cell treatment. Experimental *in vitro* and *in vivo* studies indicated improved T-cell activity for combined HMA or HDAC inhibitors with CD33-, CD123-, and CD70-directed CAR-T cells or bispecific antibodies (126, 164–166).

Multiple lines of evidence support the view that epigenetic silencing of NKG2D-ligands (NKG2DL) contributes to impaired NK-cell function, which was reversed with HMA treatment in studies on cultured NK cells (167–169). In preclinical AML models, decitabine enhanced the activity of BI836858, an anti-CD33 antibody that also engages NK cells via CD16 (170). In contrast, combining the NK-cell engaging and CD123 targeting monoclonal talacotuzumab with decitabine could not improve responses over decitabine alone in a phase II/III trial (171). Based on these data, combinations of HMAs with bispecific antibodies or CAR-T/CAR-NK cell treatment may also constitute an attractive combination. A comprehensive assessment of the biological effects of HMAs on cellular treatments is required before these combination treatments can be introduced into clinical testing.

Combining the more selective second-generation targeted epigenetic drugs with cancer immunotherapy appears attractive as it may be associated with fewer unintended effects and more efficacy. However, it also requires detailed studies before those concepts enter clinical trials. In particular, more data are needed on how these individual compounds may modulate effector and regulatory immune cell function in the context of substance-specific effects in leukemia cells. Most selective epigenetic compounds, for example, IDH or menin inhibitors, alter specific gene expression and induce differentiation (54, 55, 84, 93, 94), (**Figure 1**). These effects may represent a synergistic opportunity for combinatorial approaches as they commonly lead to the induction of surface antigen expression that may be utilized for immunotherapy, as reported with other targeted agents (172). Several other compound-specific effects may confer synergy with immunotherapeutic approaches: BET inhibitors, for instance, have been reported to impair PD-1 expression and T-cell exhaustion *in vitro* (173). Accordingly, improved T-cell expansion and anti-tumor efficacy have been observed in an adoptive T-cell transfer model upon JQ1 treatment (174). In a landmark study, it was observed that LSD1 inhibition stimulated T-cell-mediated anti-tumor responses by inducing endogenous ERV expression in cancer cells that resulted in type 1 interferon activation (175). Confirmative studies are needed before these approaches can be translated into clinical applications.

## Summary and outlook

As outlined above, immunotherapy has dramatically improved treatment outcomes in patients with many cancers while these approaches have been far less successful in AML.

While the detailed mechanisms behind the relative resistance against immunotherapy remain obscure, the low immunogenicity of myeloid blasts for immune checkpoint blockade (31, 33, 34) and the difficulties in defining AML-specific antigens not expressed on HSCs for immune-directed treatment (32, 119, 120) remains an unsolved challenge. Epigenetic manipulation was shown to improve the responses to immunotherapy by inducing neoantigens, increasing antigen presentation, and co-regulating immune checkpoints (35, 36, 96, 141–144, 146–148). Clinical trials evaluating the combination of non-selective epigenetic drugs (such as HMAs) with checkpoint inhibitors have mainly reported modest activity in the R/R AML setting (12, 153, 176), while approaches combining the anti-CD47 antibody magrolimab with azacitidine with or without venetoclax resulted in very promising response rates in clinical trials (162, 177). Clinical data for the combination of HMAs with cellular immunotherapy is pending, while CAR-NK cell concepts seem auspicious due to their only temporary toxicity for the normal hematopoiesis (129). Promising strategies include the introduction of (second-generation) targeted epigenetic drugs into immunotherapeutic treatment regimens. These drugs commonly have less adverse effects and their common ability to release the differentiation block in AML blasts accompanied by antigen-induction may enhance cellular immunotherapy. Studies that define specific effects of these drugs on various immune cells are underway to enable translation of these concepts into clinical investigation.

## Author contributions

JR: Writing – original draft, Writing – review & editing. EU: Writing – review & editing. MK: Writing – review & editing, Writing – original draft.
